# Mild hyperthermia induced by gold nanorods acts as a dual-edge blade in the fate of SH-SY5Y cells via autophagy

**DOI:** 10.1038/s41598-021-02697-y

**Published:** 2021-12-14

**Authors:** Maryam Ghafarkhani, Cigir Biray Avci, Reza Rahbarghazi, Abbas Karimi, Majid Sadeghizadeh, Amir Zarebkohan, Farhad Bani

**Affiliations:** 1 Department of Medical Nanotechnology, Faculty of Advanced Medical Sciences, Tabriz University of Medical Sciences, 516661-4733 Tabriz, Iran; 2 Department of Medical Biology, Medical Faculty, Ege University, Bornova, 35100 Izmir, Turkey; 3 Department of Applied Cell Sciences, Faculty of Advanced Medical Sciences, Tabriz University of Medical Sciences, Tabriz, Iran; 4 Stem Cell Research Center, Tabriz University of Medical Sciences, Tabriz, Iran; 5 Department of Molecular Medicine, Faculty of Advanced Medical Sciences, Tabriz University of Medical Sciences, Tabriz, Iran; 6 Department of Nanobiotechnology, Faculty of Medical Sciences, Tarbiat Modares University, Tehran, Iran

**Keywords:** Cancer, Oncology, Nanoscience and technology

## Abstract

Unraveling unwanted side effects of nanotechnology-based therapies like photothermal therapy (PTT) is vital in translational nanomedicine. Herein, we monitored the relationship between autophagic response at the transcriptional level by using a PCR array and tumor formation ability by colony formation assay in the human neuroblastoma cell line, SH-SY5Y, 48 h after being exposed to two different mild hyperthermia (43 and 48 °C) induced by PTT. In this regard, the promotion of apoptosis and autophagy were evaluated using immunofluorescence imaging and flow cytometry analyses. Protein levels of Ki-67, P62, and LC3 were measured using ELISA. Our results showed that of 86 genes associated with autophagy, the expression of 54 genes was changed in response to PTT. Also, we showed that chaperone-mediated autophagy (CMA) and macroautophagy are stimulated in PTT. Importantly, the results of this study also showed significant changes in genes related to the crosstalk between autophagy, dormancy, and metastatic activity of treated cells. Our findings illustrated that PTT enhances the aggressiveness of cancer cells at 43 °C, in contrast to 48 °C by the regulation of autophagy-dependent manner.

## Introduction

Nano-bio interactions and biological consequences of using nanotechnology-based therapies have been considered a hot topic in the recent decay because of its importance in the bench to bedside translation. Considering various advantages such as noninvasive entity, short treatment schedule, lower side effects to healthy cells, and high efficiency compared to chemo/radiotherapy, PTT has attracted immense interest in cancer therapy^1–3^. PTT can selectively increase the intracellular and/or tumor niche temperature to the range of 40–80 °C. These features alter and destroy cell membrane-bounded proteins, cellular skeleton scaffold, and subcellular organelles, thereby leading to tumor cell degeneration and attrition^4^.

On the other hand, it was confirmed that cancer cells could evade chemotherapeutic agents and therapeutic strategies by engaging biological mechanisms like autophagy^5–8^. Autophagy is a multistep lysosomal degradation pathway that mediating the removal of damaged cellular entities in response to various insults^9–11^. Importantly, autophagy promotion is completely associated with the intensity of stress plays a dual role in cancer fate, as promoter or suppressor^12–15^. So, modulation of autophagic response is considered a promising strategy to improve cancer therapy in clinical trials^13,16^.

Regarding the significant impact of stress on tumor dynamic growth and expansion, it is important to know which kind of cell death mechanisms will occur inside the cancer niche post-PTT^5^. For example, sudden release of large amounts of growth/proinflammatory factors following necrotic death increases the risk of exacerbating the aggressiveness and metastatic capability of the remaining cancer cells^5,17^. Commensurate with these comments, researchers have focused on mild hyperthermia (43–48 °C) induced by PTT, to avoid such risks^18–20^.

To our knowledge, the exact and close relationship between tumor cell death type induced by PTT and tumorigenesis capability of cancer cells has not yet been completely addressed^17,21^. More recently, Yujuan and co-workers noted the initiation of necrotic and apoptotic changes in tumor cells is closely associated with temperature variation during PTT^3^. In an address to this issue, researchers have shown that PTT can induce autophagy in several studies^22,23^. They have also found that the role of autophagy in PTT is protective, so they suggested that autophagy inhibitors (HCQ, 3-MA) can increase PTT efficiency as an auxiliary treatment for PTT^24,25^. But the main questions here are whether the level of autophagy can lead to dormancy or increase the tumorigenesis ability of cells or not? Will the use of autophagy inhibitors at any level of autophagy induction increase the effectiveness of PTT? So, the PTT's major challenge is somehow controlling the exact parameters of PTT like the temperature inside the cancer cells or the tumor mass, near-infrared (NIR) irradiation time, and power of the laser for dictating the cancer cells to clean death.

**Figure 1:**
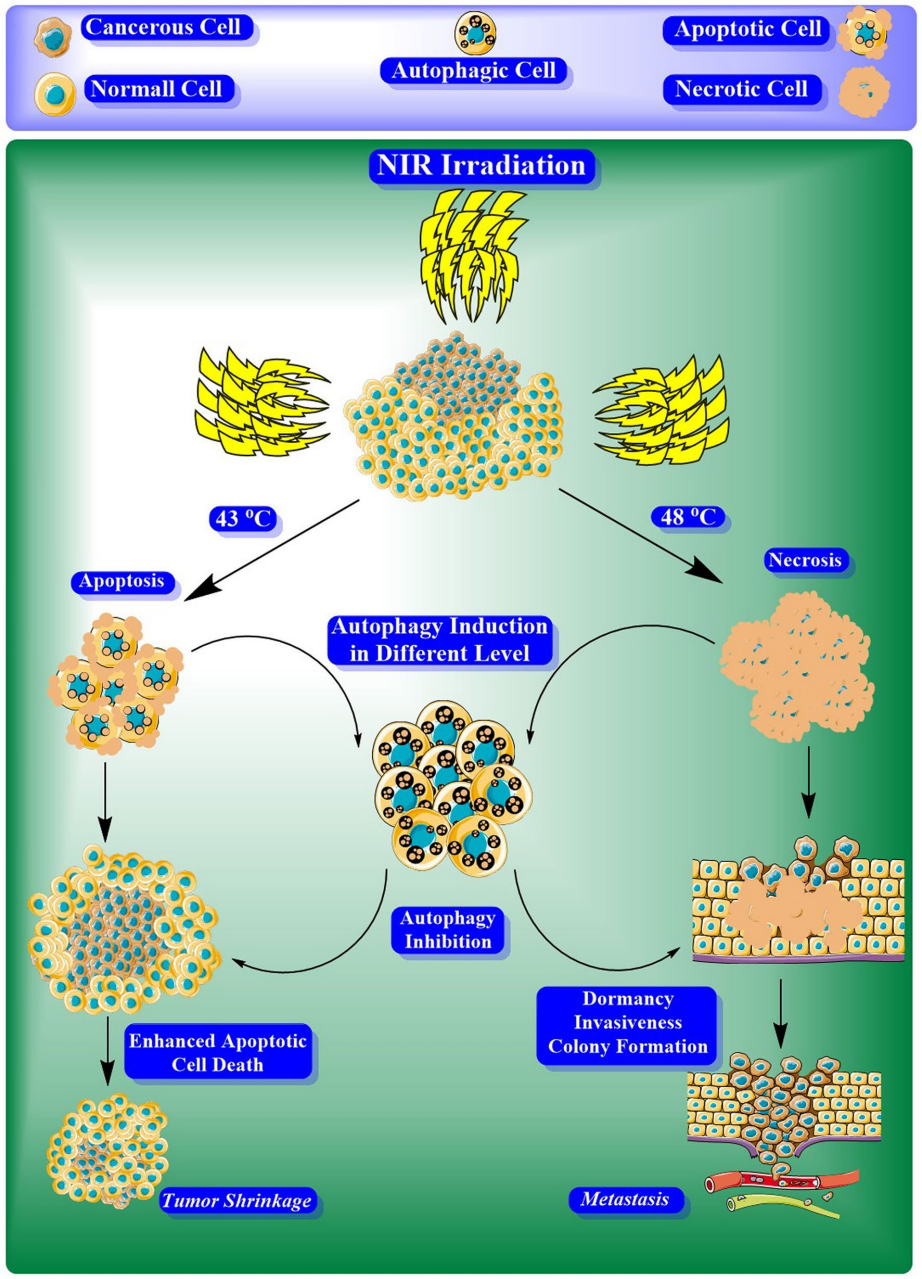
The concept of this study. May photothermal therapy invoke the tumorigenesis of cancer cells unwantedly by activating the autophagy pathway?

According to the started clinical trials in human prostate cancer-bearing patients^26^, and considering the risks mentioned above and the lack of sufficient information, it seems that the unraveling of cancer cells' fate in response to different PTT regimes is indisputable. In the current experiment, we investigated the modulation of autophagy response and its final effects on tumorigenesis ability in human neuroblastoma cell line SH-SY5Y after exposure to lower and higher temperatures (in the range of mild hyperthermia) produced by albumin-coated gold nanorods (AuNRs)-mediated PTT. The results of this study could help us develop efficient modalities in favor of cancer inhibition via PTT. For better understanding of our hypothesis, we prepared an illustration which is referred as Fig. 1.

## Results

### Synthesis, modification, and cellular uptake efficiencies of BSA–GNRs

**Figure 2:**
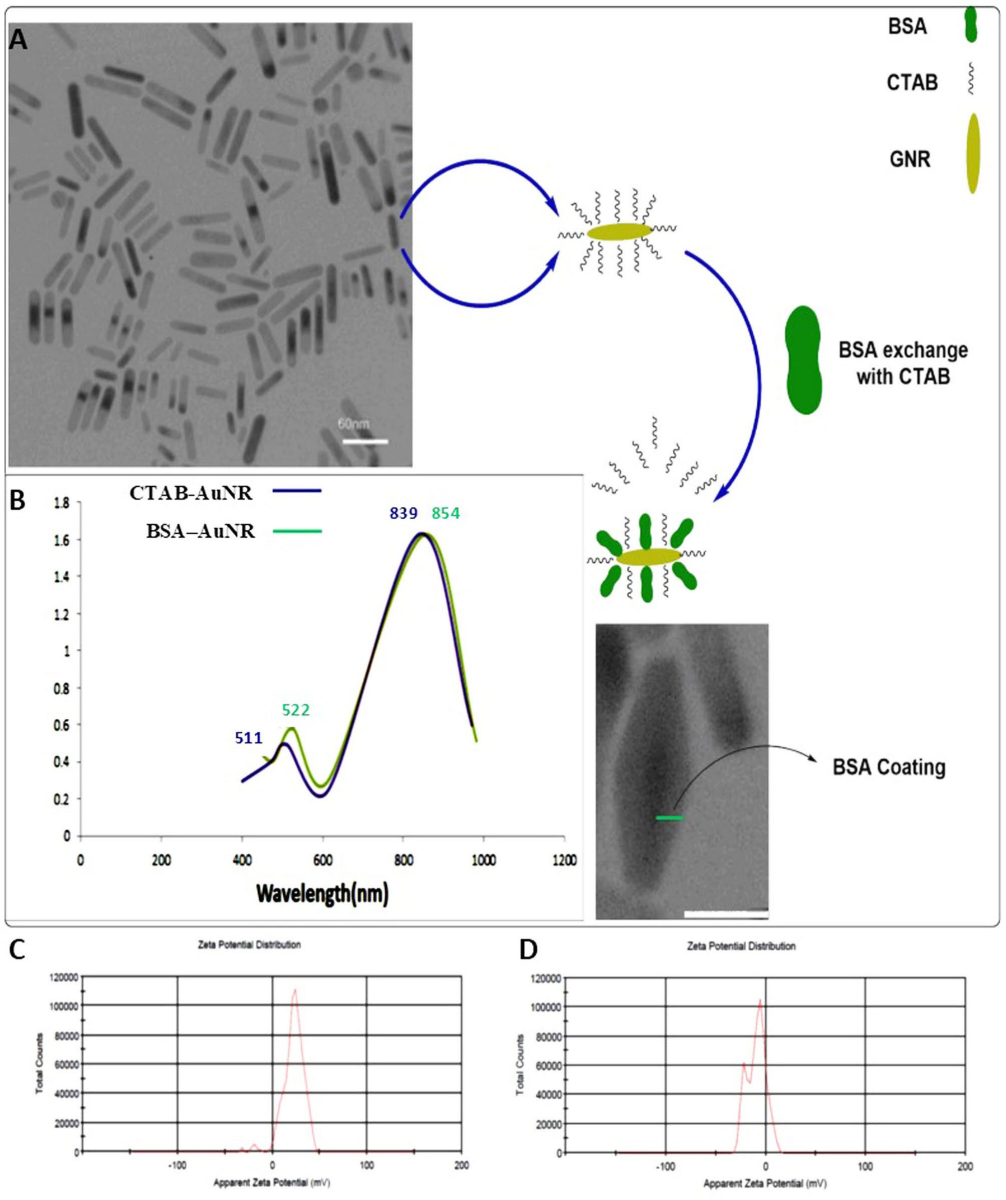
Characterization and performance monitoring of synthesized AuNRs. (**A**) Structural illustration and TEM image of AuNRs and BSA–AuNRs. (**B**) UV–Vis absorption spectra of CTAB–AuNRs and BSA–AuNRs. (**C**) Zeta potential of CTAB–AuNRs and (**D**) BSA–AuNRs were measured using DLS.

In this study, AuNRs were developed by a seedless growth technique that produced CTAB capped nanorods (CTAB–AuNRs)^26^. TEM imaging showed monodispersed rod-shaped AuNRs with an average of 26.4 nm in length and 7 nm in width (Fig. 2A). Additionally, UV–Vis absorption spectra of AuNRs exhibited peak SPR frequency at ~ 511 nm, and we tuned the longitudinal peak SPR frequency to a sharp peak of ~ 839 nm, by modifying the aspect ratio to 3.77 (Fig. 2B).

DLS was employed to measure AuNRs size, zeta potential, and stability in aqueous solutions. Results showed that the CTAB–AuNRs had an average surface charge of + 22 mV with an average hydrodynamic diameter of 32.1 ± 1.3 nm (Fig. 2C). Functionalization (conjugation) of the AuNRs was performed through the exchange of CTAB by BSA to generate the BSA–AuNR complex. According to our data, BSA molecules were efficiently attached to the surface of AuNRs, which was indicated by the redshift of the plasmon peak of AuNRs in the UV–Vis spectrum (Fig. 2B), and the change of surface charge (zeta potential) analyzed by DLS (Fig. 2D). The UV–Vis spectra showed a peak at maximum absorbance around 854 nm for BSA–AuNRs. After BSA conjugation, the hydrodynamic size of BSA–AuNRs was slightly larger than that of CTAB–AuNRs, and BSA–AuNRs surface charge reached − 9.22 mV (Fig. 2D). According to these data, the BSA–AuNR complex could be well stored in PBS solution and resuspended in a culture medium without aggregation for days. The above data suggested that BSA–AuNRs were suitable candidates for biological applications.

To test the cellular uptake of the BSA–AuNRs, we exposed the human neuroblastoma cell line, SH-SY5Y cells, to the BSA–AuNR complex for 4 h. Based on our results, the efficiency of cellular uptake of BSA–AuNR nanoparticles was 15%.

### The photothermal conversion efficiency of BSA–AuNRs

**Figure 3:**
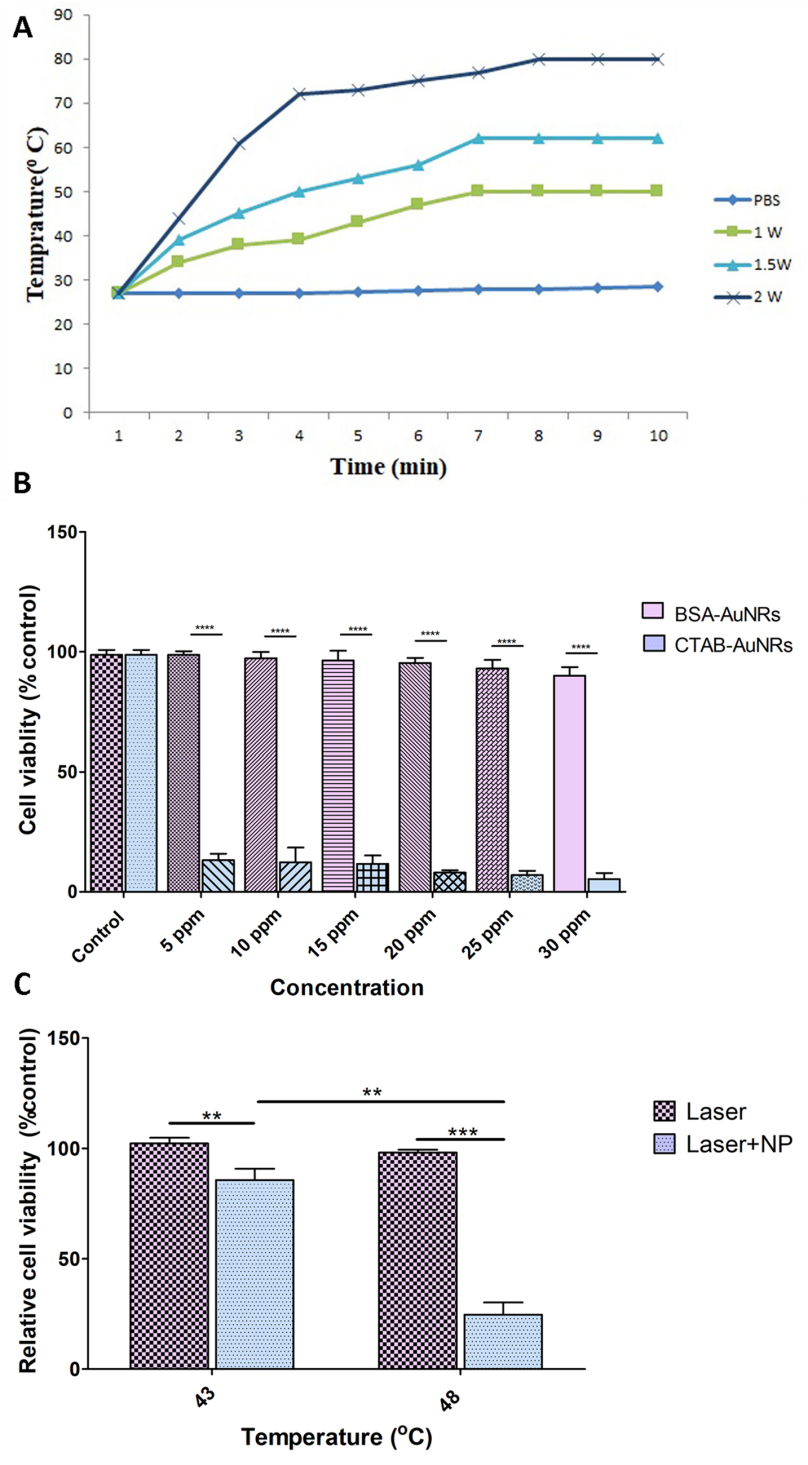
Modeling of temperature elevation by PTT and MTT assay of nanoparticles. (**A**) The photothermal profiles of pure water and aqueous dispersions of BSA–AuNRs with 30 ppm of BSA–AuNRs examined using 808-nm laser irradiation with a different power density of 1.4 and 2 W/cm^2^. (**B**) Cell viability of CTAB–AuNRs and BSA–AuNRs at different concentrations (5–30 ppm). (**C**) Relative cell viability in response to 808-nm laser irradiation with a power density of 2 W/cm^2^ after incubation with or without 30 ppm of BSA–AuNRs cell culture media. One-Way ANOVA and Tukey post hoc analysis. (n = 3) **P < 0.01; ***P < 0.001; ****P < 0.0001.

To test whether BSA–AuNRs can introduce efficient photothermal effects, the temperature of BSA–AuNRs aqueous solution was recorded upon NIR irradiation using a digital thermometer while PBS-free nanoparticles were used as a control group (Fig. 3A). Upon excitation by incident radiation of appropriate wavelength, BSA–AuNRs generated sharp local heating by the photothermal conversion of the absorbed light energy, rendering these particular particles as extremely efficient “nano-heaters”. Irradiating 4.5 ppm (uptaken nanoparticles) aqueous solution of BSA–AuNRs with a continuous wave laser (808 nm) for 8 min with 1.4 W/cm^2^ power of laser resulted in a rise in temperature from 27 to 50 °C (ΔT = 23 °C); however, when same solutions were irradiated with power densities 2 W/cm^2^, the temperature of the solution increased from 27 to 80 °C (ΔT = 53 °C) (Fig. 3A). Monitoring temperature change (ΔT) exhibited a rapid rise (ΔT > 7 °C) within the first minute of irradiation, followed by a saturation trend (ΔT < 1 °C) after 4 min.

### BSA–AuNRs plus PTT decreased viability of human SH-SY5Y cells

The safety and biocompatibility of BSA–AuNRs were investigated on SH-SY5Y cells using MTT assay and resulted compared to the CTAB–AuNRs. Figure 3 showed cell viabilities of SH-SY5Y treated with different concentrations of AuNRs (5, 10, 15, 20, 25, and 30 ppm) decorated with CTAB and BSA after 24 h. The precise concentration of AuNRs in the CTAB–AuNRs and BSA–AuNRs complexes was calculated by AAS. The incubation of cells with CTAB–AuNRs promoted significant cytotoxicity at all concentrations (5–30 ppm) (Fig. 3B). At the same time, the replacement of CTAB with BSA increased the survival rate and closed to the near-to-control levels. Of note, the SH-SY5Y cell viability increased from 8 in the CTAB–AuNR-treated group (20 ppm) to 95.33% in the BSA–AuNRs (20 ppm) group (P < 0.0001). MTT assay showed that 90% of cells were viable even at the highest concentration of BSA–AuNRs (30 ppm) after 24 h (Fig. 3B, P < 0.0001).

Next, the viability of SH-SY5Y cells was investigated after treatment with BSA–AuNRs and PTT. To this end, cells were treated with 30 ppm BSA–AuNRs and irradiated with an 808-nm NIR laser, after 4 h incubation and 3 times washing with cold PBS. We noted that the temperature of groups increased up to 43 and 48 °C eight minutes after exposure to laser intensities 0.3 and 0.9 W, respectively. Interestingly, data revealed the reduction of survival rate 85.6 to 24.6% compared to the control group as the temperature of the sample increased from 43 to 48 °C (Fig. 3C, P < 0.01). These results suggested BSA–AuNRs have superior PTT efficacy and excellent biocompatibility in vitro.

### Treatment of SH-SY5Y cells with BAS–AuNRs plus PTT induced apoptotic and necrotic cell death

**Figure 4:**
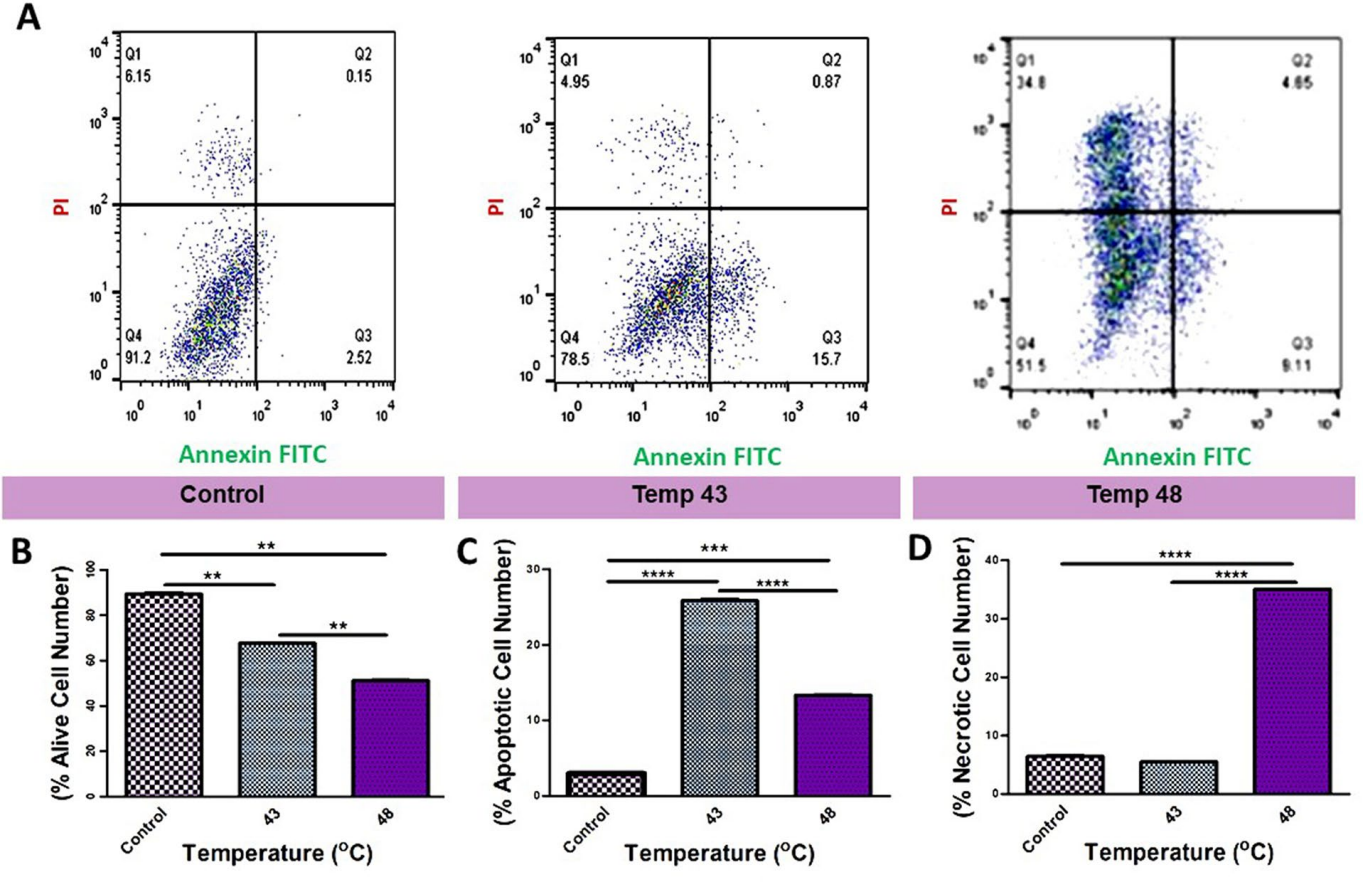
FITC-labeled annexin V/PI apoptosis assay. (**A**) Flow cytometry evaluation of apoptosis in different groups; Control, 43, and 48 °C (from left to right). (**B**) The number of alive cells after PTT induction between the mentioned groups (P < 0.01). (**C**) The number of apoptotic cells after PTT induction between the abovementioned groups (P < 0.0001). (**D**) The number of necrotic cells after PTT induction in the treated groups (P < 0.0001). One-Way ANOVA and Tukey post hoc analysis (n = 3). **P < 0.01; ***P < 0.001; ****P < 0.0001.

To further evaluate the cell death mechanism, cells with different treatments were stained with Annexin V/FITC and PI and then processed by flow cytometry. As shown in Fig. 4A, neither early apoptosis nor late apoptosis was detected in the control and BSA–AuNRs (data not shown) groups. Treatment of cells with the combination of BSA–AuNRs and PTT induced apoptotic changes (early apoptotic cells: 20.1%; late apoptotic cells: 5.43%) necrotic cell death (18.2%) at 43 °C (Fig. 4C,D). According to our data, we showed a significant increase in the number of necrotic cells at higher temperatures (48 °C) compared to groups 43 °C (Fig. 4D, P < 0.0001). These results revealed that the increase of intracellular temperature under PTT could change the cell death type. It seems that higher temperatures induced rapid necrotic cell death rather than slow cell death apoptosis. Taken together, PTT effectively exerts tumoricidal effects on human SHSY-5Y cells, and the type of cytotoxicity is closely associated with ambient temperature (Fig. 4B–D).

### Assessment of autophagic response by flow cytometry assay

**Figure 5:**
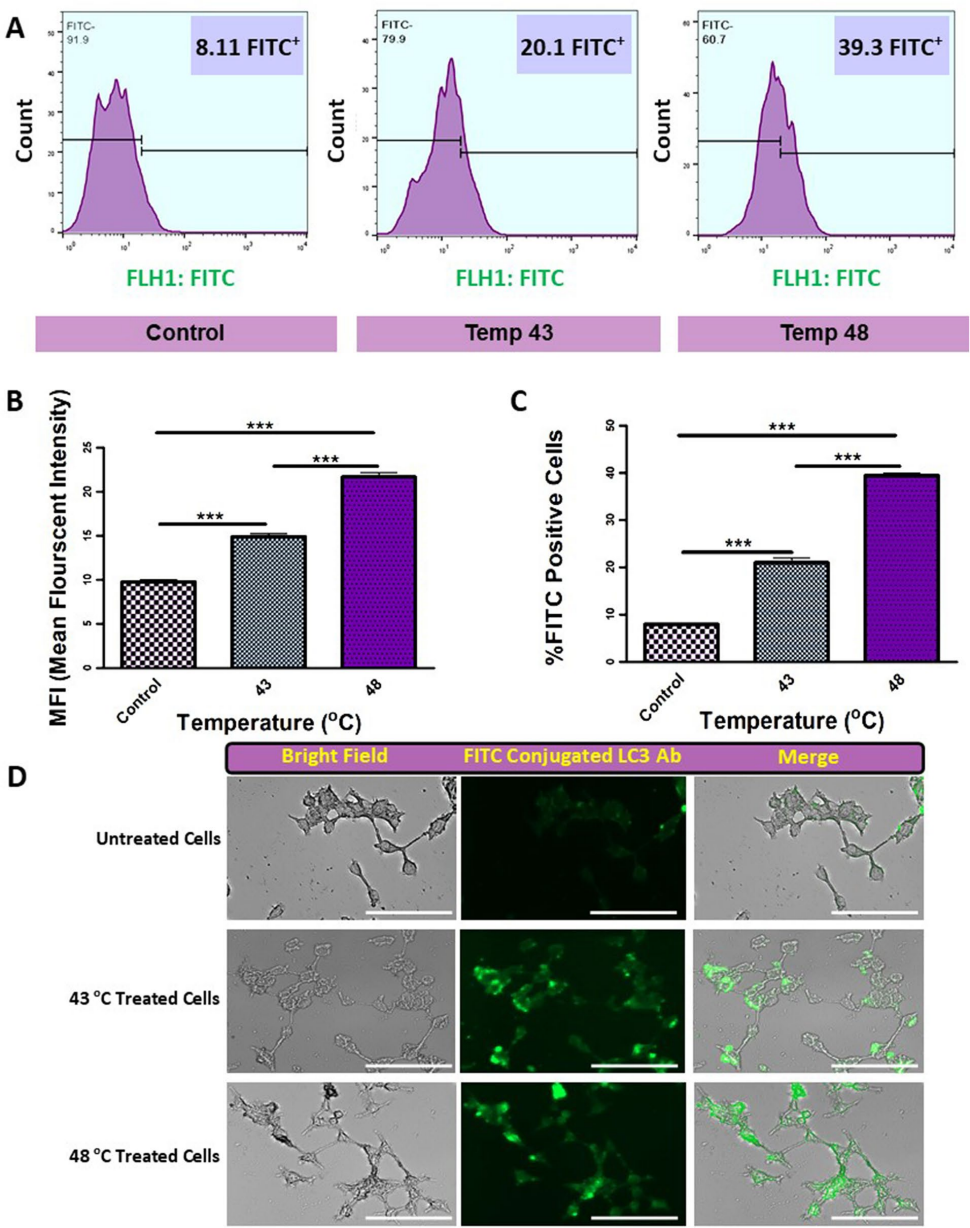
Quantitative and qualitative evaluation of autophagy induction. (**A**,**C**) Flow cytometry histogram of treated cells in different groups. As were showed in this section, the percentage of positive FITC-conjugated LC3 antibody cells increased from left to right by increasing the induced temperature (P < 0.001). (**B**) The mean fluorescent intensity of treated groups. Our results showed that in addition to the number of FITC-labeled LC3 antibody-positive cells, the intensity of signals significantly increases by temperature elevation (P < 0.001). (**D**) Qualitatively evaluation of autophagy by fluorescent microscopy. It was showed that the autophagy levels in the cells increase by induction of PTT. One-Way ANOVA and Tukey post hoc analysis (n = 3). ***P < 0.001.

The autophagic response was also monitored using flow cytometry analysis. Data showed that the percent of apoptotic cells was not statistically significant in cells treated with BSA–AuNRs compared to the non-treated group (Fig. 4B). By increasing the temperature from 43 to 48 °C (Fig. 5A), the number of apoptotic cells expressing LC3 was also increased. Despite an increase in the number of LC3-positive cells in groups exposed to the combination of BSA–AuNRs and PTT, these changes were not significant. Also, our findings show that in addition to the increase of autophagic cell percentage, the amounts of LC3 increase in response to the temperature elevation (Fig. 5B,C). Besides, we confirmed these findings qualitatively (Fig. 5D). These data showed that the treatment of human cancer cells with BSA–AuNRs plus PTT could alter the number of cells entering apoptosis.

### Exposure of AuNRs to PTT changed the expression of autophagy machinery effectors in a temperature-dependent manner

**Figure 6:**
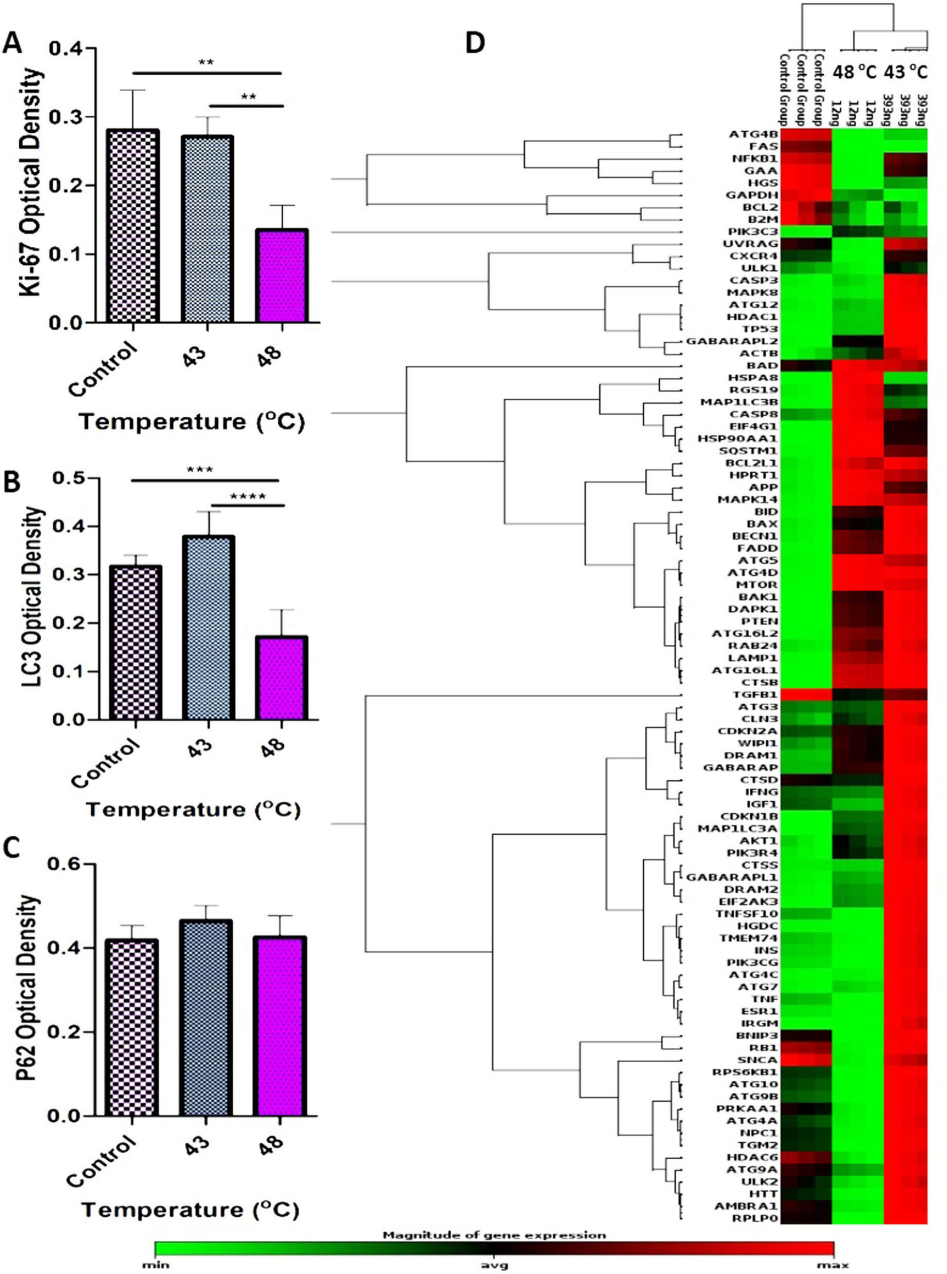
Protein level of Ki-67, P62, and LC3 measured by ELISA and PCR array of 86 genes involved in autophagy machinery. (**A**) Ki-67 protein level (as a proliferation indicator), significantly decreased followed by 43 and 48 °C, which shows the reverse effect of temperature elevation on the cell’s proliferation ability (P < 0.01). (**B**) LC3 protein level (as autophagy indicator) showed different behavior of cells in response to temperature elevation (P < 0.0001). (**C**) Interestingly, our results showed that the P62 protein level does not change significantly in response to temperature increase. (**D**) Phylogenic tree of 86 genes related to autophagy. Our results showed that 54 genes from those changed in response to the PTT. One-Way ANOVA and Tukey post hoc analysis (n = 3). **P < 0.01; ***P < 0.001; ****P < 0.0001.

To evaluate the effect of temperature on certain autophagy responses in human SH-SY5Y cells, the expressions of autophagy-related genes were monitored in two temperatures (43 °C and 48 °C) after treatment with the combination of AuNRs and PTT by RT^2^ Profiler PCR Arrays-Human Autophagy^27^. Our findings showed that in a lower and higher level of mild temperature hyperthermia (43 °C and 48 °C) significantly increased the expressions of several autophagy-related indicators in the SH-SY5Y cell line compared to the control group (Fig. 6D). The results of the transcriptomic analysis showed a significant change in the expression of effectors playing a critical role in different components of autophagy machinery like chaperone-mediated autophagy (CMA) and macroautophagy (Fig. 6D, Supp. Data).

#### Macroautophagy machinery components

The autophagy regulating machinery is composed of different genes that control three distinctive processes, including macroautophagy, microautophagy, and CMA^28^. Through the invagination of the lysosomal membrane, microautophagy controls the internalization of cytosolic recycled proteins and organelles^29,30^. Of 86 genes associated with autophagy, the expression of 54 genes was changed in response to temperature fluctuation induced by PTT protocol (Fig. 6D, Supp. Data). According to our data, the expression of most genes was up-regulated at 43 °C and 48 °C. It seems that the intensity of these changes was different between 43 and 48 °C groups (Fig. 5D). The association of these effectors with other signaling pathways seems to dictate specific cell behavior.

#### Vacuole formation, induction, and nucleation

Vacuole formation, vacuole targeting, ubiquitination, and autophagosome–lysosome linkage are the most important components of macroautophagy^31–33^. The autophagy-related genes (ATG) have an indisputable role in controlling the autophagosome formation and macroautophagy processes^34^. As shown in Fig. 6, the expression profile of some genes [Becn1 (BECN), Ulk1 (the Unc-51 like kinase 1 (*C. elegans*)], RGS1, GABARAP, Atg9a, Atg4a, and ATG12 involved in the vacuole formation were not significantly changed in groups 43 °C and 48 °C in comparison with the control group (Supp. Data). On the contrary, the up-regulation of ATG16L1 (1364.50-fold vs. 1164.10-fold), ATG4d (6.43-fold vs. 6.52-fold), ATG5 (2.63-fold vs. 2.94-fold), GABARAPL2 (3.22-fold vs. 2.12-fold), MAP1LC3A (LC3-I: 12.25-fold vs. 5.05-fold), MAP1LC3B (LC3-II: 2.05-fold vs. 4.58-fold) was indicated at 43 °C and 48 °C, respectively. We found that the expression of two genes is different in human cancer cells exposed to different temperatures. Contrary to the results of group 43 °C, both genes AMBRA1 (− 2.05-fold) and ATG9B (− 4.07-fold) were down-regulated at 48 °C. The exposure of human SH-SY5Y cells up-regulated ATG9B (2.44-fold) and IRGM (46.37-fold) at 43 °C. We found down-regulation of ATG4B in both 43 °C (− 3.28-fold) and 48 °C (− 5.08-fold) groups compared to the non-treated control (Fig. 6D, Supp. Data).

#### Ubiquitination and phagophore expansion

Two ubiquitin-like systems, including LC3 conjugation and Atg12–Atg5–Atg16L, are crucial partners for the elongation of the pre-autophagosomal structure (PAS) system^35^. Data showed that except Atg12, Atg5 and Atg16L were up-regulated in the neuroblastoma cell line during exposure to 43 °C (Atg5: 2.63-fold and Atg16: 2.94) and 48 °C (Atg5: 1346.50-fold and Atg16: 1164.10-fold) (Fig. 6D, Supp. Data). These two systems work in two different manners. By an enzymatic function of Atg7 and Atg10, the protein Atg12 is attached to the Atg5 factor. After that, the Atg12–Atg5 complex binds non-covalently to the Atg16L protein, and this complex serves as an e3-like ubiquitin ligase for autophagosomal membrane formation and development^34^.

Secondly, Atg7 and Atg3 cooperatively connect another ubiquitin-like protein (LC3) with phosphatidylethanolamine (PE) to increase the intracellular concentration of LC3II. LC3-II is touted as a final mediator which participates in the elongation phase of the autophagosome^36^. In the SH-SY5Y cells treated with mild hyperthermia, the expression level of LC3-I and MAP1LC3B was up-regulated at 43 °C and 48 °C. However, the intensity of expression varied in both groups. This different but aligned pattern in the expression of LC3 confirmed the fact that autophagy could be stimulated at different temperatures with a certain activity. These features lead to different consequences and behavior in cancer cells^37^. Another important gene that belongs to autophagy machinery, HDAC6 (Histone deacetylase 6), is crucial for ubiquitin-based control of autophagy and facilitating the lysosome-autophagosome attachment^38^. The combination of AuNRs and PTT did not alter the expression of HDAC6 at 43 °C and 48 °C groups compared to the control (Fig. 6D, Supp. Data).

#### Vacuole targeting and autophagosome–lysosome linkage

The next step after autophagosome formation is tethering and merging the autophagosome with lysosomal vacuole and, subsequently, the release of autophagic vesicle contents into the lysosomal inner space^39^. In the following steps, the contents are degraded by lipases and hydrolases in the lysosomes. Finally, the products of degradation processes are released into the cytosol by efflux transporters^40^. The transcriptome profile of some associated proteins involved in the fusion process has been evaluated in this study. Lamp1, the Lysosomal-associated membrane protein 1 gene, was significantly up-regulated at 43 °C (3.64-fold) and 48 °C (3.08-fold), respectively. In contrast, Atg4b was significantly down-regulated 43 °C (− 3.28-fold) and 48 °C (− 5.08-fold). Atg4c, another autophagosome–lysosome linkage-related gene, was up-regulated (3.31-fold) at 43 °C, while the treatment of cells at 48 °C did not alter transcription level compared to the control (Fig. 6D, Supp. Data). The expression of GABARAP, the Gamma-aminobutyric acid (GABA) A receptor-associated protein-like, was not changed at both temperatures. Concerning these findings, it seems that the autophagosome–lysosome linkage step of autophagy was stimulated more effectively at 43 °C rather than 48 °C.

#### Chaperone-mediated autophagy

CMA is a specific pathway that cells used directly for lysosomal degradation of assembled proteins without any lysosomal membrane changing following intra/extracellular stresses^35^. CMA signaling can be controlled by some of the heat shock proteins like 71 kDa protein (Hsc70), also known as HSPA8, an important member of the heat shock protein 70 family (Hsp70). HSPA8, in combination with HSP90AA1 (cytosolic class A member 1 co-chaperones) forms chaperone/substrate complex. This complex uses a pentapeptide motif (KFFRQ) targeted misfolded cytosolic proteins^41^. In our study, the expression profile of HSP90AA1gene was not significantly changed in response at 43 °C compared to 48 °C. This finding shows that PTT parameters used in this study cannot properly alter the expression of HSP90AA1. Interestingly, the expression of HSPA8 is up-regulated only at 48 °C (5.45-fold). These results show that CMA is activated at 48 °C (Fig. 6D, Supp. Data).

### The activation of autophagy by PTT alters the colorogenic capacity of human SH-SY5Y cells

**Figure 7:**
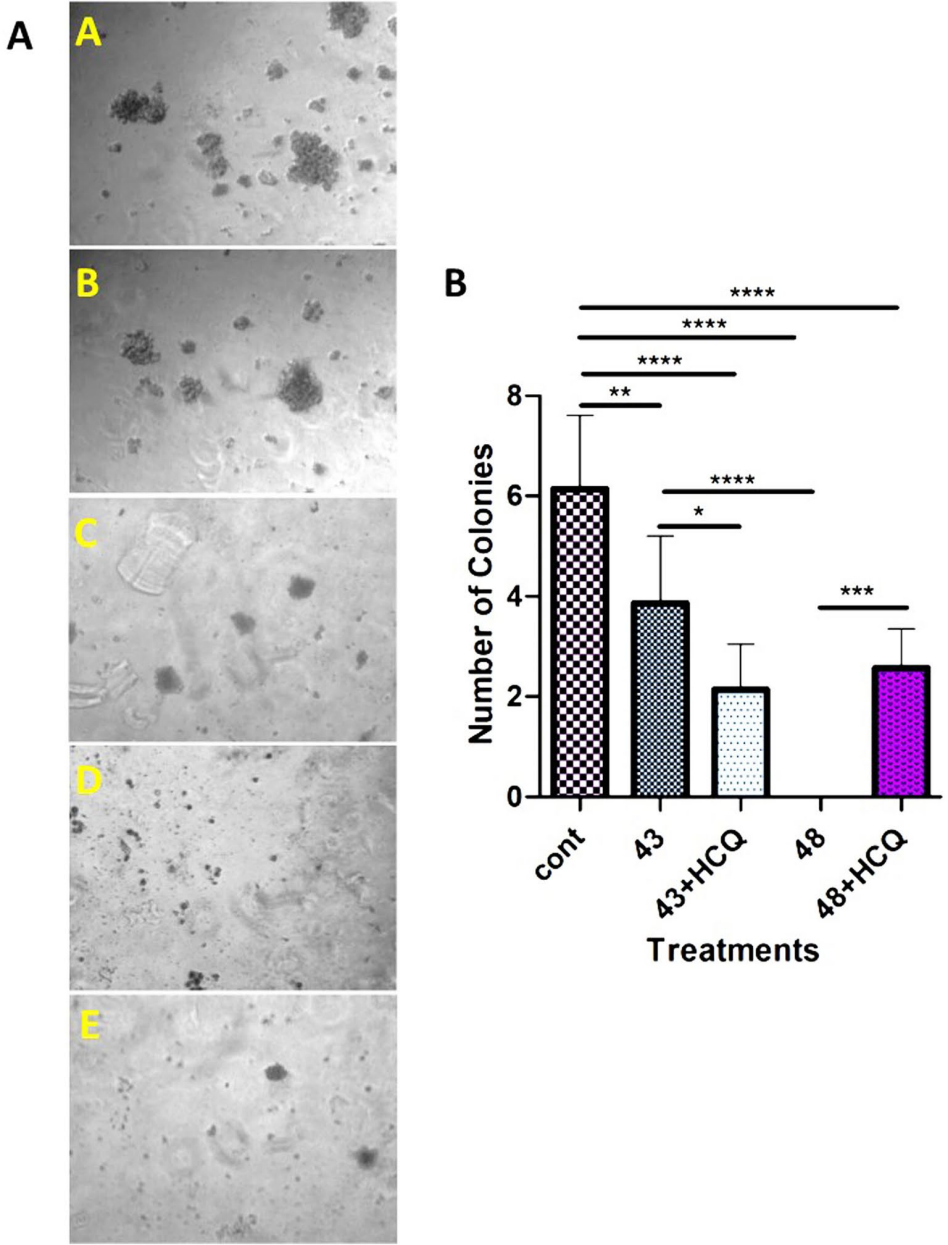
Tumor formation assay. (**A**) A–E images show the colony formation ability in response to two different PTT regimens, in the presence and absence of HCQ, qualitatively. (**B**) Statistical analysis of colony formation assay shows that human neuroblastoma cell line behavior completely different in response to 43 and 48 °C. In the former, autophagy has a protective role in cancer cells fate (P < 0.05), while in the latter, autophagy exerts a cytotoxic effect on cancer cells (P < 0.001). One-Way ANOVA and Tukey post hoc analysis. (n = 3). *P < 0.05; **P < 0.01; ***P < 0.001; ****P < 0.0001.

In this study, we used the colony formation assay to investigate the PTT’s effect at two different temperatures, 43 °C, and 48 °C, on human SH-SY5Y cells. Our results showed that incubation of cells at 43 °C had protective effects on the cancer cells, while the promotion of autophagy at 48 °C led to cytotoxicity in SH-SY5Y cells (Fig. 7A). To show the protective effect of autophagy in SH-SY5Y cells after being exposed to PTT, we used an autophagy inhibitor, 10 µM HCQ. We noted that the incubation of cells with HCQ sensitized them to the detrimental effect of PTT, which was blunted by the activation of autophagy. Data confirmed that the number of colonies was significantly decreased in the presence of HCQ after being exposed to 43 °C. This finding suggests that autophagy stimulation by PTT at 43 °C leads to increased SH-SY5Y cell survival, indicating the protective effect of autophagy in cancer cells exposed to mild hyperthermia. The inhibition of the final phase of autophagy by HCQ contributes to the accumulation of toxic metabolites and unfolded/misfolded proteins, inducing apoptosis^42^. In contrast, the exposure of cancer cells to higher temperatures, such as 48 °C destroyed many cells quickly and inhibit colorogenic capacity (Fig. 7B). These findings are consistent with the results obtained from the study of genes involved in autophagy. The changes in the expression profile of genes in response to environmental stresses confirmed, and its possible role in the alteration of cancer cell properties have been established in the last decade^43^.

The induction of autophagy can result in three types of consequences, including dormancy, invasiveness, and death^44,45^. Along with this statement, we measured the expression level of Ki-67 protein as a marker of cell division using ELISA (Fig. 6A). Based on our data, Ki-67 was not changed in cells exposed to 43 °C compared to the control. In contrast, applying 48 °C inhibited significantly the synthesis of Ki-67 and LC3 compared to non-treated control cells and 43 °C group (Fig. 6A,B). Our results showed the up-regulation of important genes involved in dormancy, including CTSB, P53, IFN-B, IFN-Y, CDKN1B (P27), CDKN1A (P21), and PTEN at 43 °C group compared to the 48 °C group (Supp. Data, Fig. 6C,D). Considering the potency of 43 °C to induce the synthesis of nuclear factor Ki-67 and stimulate colorogenic capacity and autophagic response in the SH-SY5Y cells (Fig. 6A), we could hypothesize that the incubation of cancer cells in mild temperatures could inhibit active dynamic growth but simultaneously promote autophagic response which is a compensatory response to resist again insulting stimuli. The activation of certain genes in autophagy signaling could trigger other resistance mechanisms such as dormancy in the host cells (Supp. Data). In support of this notion, the blockade of autophagic response could decrease the cancer cells' survival rate compared to control matched groups. However, it should not be forgotten that the stimulation of autophagic responses to a certain extent can be protective. The over-stimulation of autophagy effectors could alter the expression and activity of other parallel signaling pathways, such as apoptosis, as seen in the groups exposed to 48 °C. Data from the flow-cytometry analysis further supported these features (Fig. 5).

Interestingly, the expression of autophagy machinery genes related to the invasion was increased at 48 °C. Despite the loss of a large number of cells at 48 °C, cells can form colonies in the presence of HCQ (Fig. 7B). It seems that the distinct effect of temperature on the modulation of autophagy-related genes could be responsible for certain cancer cells' behavior. Consistent with our data, it has been shown that the tumor formation process that arises from the Ras pathway activation requires high levels of autophagy for survival and cell proliferation^42^.

Concerning the induction of some effectors in autophagy signaling such as MAPK14, LAMP1, eIF4E, eIF4GI, ATG16L1, CDKN1A, CDKN1B, CTSB, and GABARAPL1 and their contribution to metastasis, we conclude that some genes have a regulatory role over other counterparts. For instance, EIF4E and eIF4G translation initiation factors are involved in tumorigenesis and patient survival (Tables 1, 3). The important role of these factors in cell migration, amplification of EMT promoters, and metastasis has been demonstrated previously (Table 2). Like ARHI, some genes play a crucial role in controlling the outcome of autophagy^13,46^. It has been shown that the re-expression of ARHI can lead to autophagic cell death even in the presence of HCQ^47^.

**Table 1:** Autophagic genes involved in the tumorigenesis process VS our findings.

Gene expression changes	Cellular outcome	Effect(s)	Consequences on tumor fate	References	Our study findings	
					43 °C	48 °C
Activation of GABARAPL1	Autophagy activation	Increase ROS	Tumor suppression	^37,50^	**2.04**	NSC
	Deletion of damaged Mitochondria					
ATG5	Autophagy inhibition	Oxidative stress	Tumor suppression	^51^	*ATG5:* **2.63**	*ATG5:* **2.94**
ATG7 (deletion)		Damaged Mitochondria			*ATG7:* **2.17**	*ATG7:* NSC
Autophagy activation	ATG4D over-expression	Intracellular LC3-B/P62 accumulation	Tumor suppression	^52^	**6.43**	**6.52**
		Autophagosome formation abortion				
Atg4B (over-expression)	Autophagy inhibition	LC3-PE degradation	Tumor suppressive	^53^	− **3.28**	**5.08**
		LC3 sequestration in the cytosol				
DNA damage	Atg4a	Autophagy activation	Tumor suppression by p53-mediated apoptosis	^54^	NSC	**1.37**
	Atg4c				**3.31**	NSC
	p53 (activation)				**3.24**	NSC
UVRAG up-regulation	Autophagy deficiency	–	Tumor suppression	^55^	NSC	**2.21**
Autophagy deficiency	ATG5 and ATG12 deficiency	Decreased survival capacity to metabolic stress	Tumor suppression (Decrease in colonization and survival capability)	^56^	**2.63**	**2.94**
					NSC	NSC
Dormancy activation	P53 overexpression induced by Cdkn1b	Pentose phosphate pathway destruction	Cell death	^57^	**3.24**	NSC
		Increased ROS	Dormancy induction by IFN-b			
ER stress	K-RAS dependent Eif2ak3^−/−^ MEFs	The decrease in VCIP and PDGFRB (angiogenic stabilizer)	Tumor suppression	^58^	**5.88**	**2.20**
			ECM destruction			
			Vast hemorrhage			
Autophagy manipulation	eIF4E/eIF4GI knockdown	The decrease in ERα, SMAD5, NFkB, CyclinD1, c-MYC, and HIF1α	The decrease in EMT promoter	^59^	**1049.1**	**1904.2**
			Increase in EMT inhibitors			
			The decrease in migration capability			
HSPA8(HSC70) complex formation	Chaperone mediated autophagy (CMA)	Cargo delivery to the lysosome	Tumor protection by autophagy	^60^	NSC	**5.45**
IFN-γ/STAT1 activation	Downregulation of Cyclin E, A, D1,2,3	Downregulation of CDK4 and CDK6	Cell cycle arrest	^61^	**2.86**	NSC
			Cancer cell dormancy			
AMBRA1	AMBRA1 role in the modulation of C-MYC phosphorylation and stability	Intracellular switch between autophagy and apoptosis	Colony formation	^62^	NSC	**2.05**
			Autophagic survival			
Inherent ATG5 or autophagy KO	These mutations may contribute to cancer development by deregulating the autophagy process	Intracellular inherent autophagy	Recurrence, Chemotherapy desensitization	^63,64^	**2.63**	**2.94**
			Increase of dormancy frequency			
CTSB	BID knockdown resulted in strong suppression of CPT-induced apoptosis and a shift of cell death towards autophagy	CTSB is one of the hub genes for dormancy	Dormancy hub gene, Survival	^65,66^	**10.97**	**9.68**
			Self-renewal			
	Increase of Beclin 1 and MAP I LC3 cellular content	CTSB and uPAR regulate the self-renewal properties of glioma stem cells	Metastasis			
CXCR4 activation	High CXCR4 expression	SDF-1α-CXCR4 signaling on autophagy induction	Promoted cancer cell survival under stress	^67^	NSC	**3.64**
	Increases autophagic activity					
FADD	FADD is also implicated in cell cycle progression, proliferation, and tumorigenesis	FADD in tumor progression via Rheb-mTORC1 pathway in breast cancer	Atg5 contributes to autophagic cell death by interacting with (FADD)	^68,69^	**4.87**	**3.59**
NFKB1	Increased activation of NF-κB and MAPK via NFKB1 deletion enhance macrophage and myofibroblast content at the repair	Cross-talk between ER stress, autophagy, apoptosis, and the NF-κB pathways controls the fate of cancer cells	NF-κB inhibition partly enhances the survival of cancer cells following BFA treatment	^70–72^	NSC	**5.84**
	Driving increased collagen deposition and biomechanical properties					
SNCA	SNCA binds to (HMGB1) blocks HMGB1-BECN1 binding, and strengthens BECN1-BCL2 binding	Deregulation of these molecular events by SNCA overexpression leads to autophagy	Tumor survival	^73–75^	NSC	**2.56**
TGM2	Putative tumor suppressor in the TP53 pathway and colony formation	Autophagy and CDKN1A-mediated cell cycle arrest	Tumor suppressor	^76^	**2.96**	NSC
HGS	Critical role in the recycling and degradation of membrane receptors	Phosphorylation of SMAD1/5/8 and TAK1/p38 to transduce BMP signaling	Tumor survival and invasion	^77,78^	**2.76**	**5.72**
	HGS-dependent TP53 exosome formation					

Significant values are in bold. *NSC* non significant change.

**Table 2:** Autophagic genes involved in invasion (colonization, proliferation, tumor formation, promotion, metastasis) VS our findings.

Gene expression changes	Cellular outcome	Effect(s)	Consequences on tumor fate	References	Our study findings	
					43 °C	48 °C
ATG5 and ATG7- RAS	Increased autophagy	Mitochondria activation	Tumor formation	^4,43^	**2.63**	**2.94**
					**2.17**	NSC
ATG16L1	Autophagy deficiency	Oxidative stress	Tumor suppression	^79,80^	**1346.5**	**1164.1**
LC3-II degradation		Damaged mitochondria				
		Inflammation induction (1β, IL-18)			**2.05**	**4.58**
Autophagy activation	p27Kip1 coaded by CDKN1B	CDK-dependent kinase inhibitor	Tumor promotion	^81^	**423.14**	**134.83**
Autophagy deactivation	ATG3/7/p62 targeting	Pfkfb3 normal expression	Tumor re-proliferation	^82^	NSC	NSC
					**2.17**	NSC
STAT1 inhibition	p27 (CDKN1B), p21(CDKN1A) upregulation	Increase in IDO1 and Kyn receptors	Tumor dormancy	^83^	**423.1**	**134.8**
		Rb hypophosphorylation	Increase in colony formation			
		Suppress E2F transcription factor activity	Decrease in proliferation			
AMBRA1	AMBRA1 role in the modulation of C-MYC phosphorylation and stability	Intracellular switch between autophagy and apoptosis	Colony formation	^84,85^	NSC	**2.05**
ATG9B mutation	Autophagy suppression	Blocked recruitment of p62-associated ubiquitinated protein for autophagosome–lysosome degradation	Tumorigenesis	^64,86^	**2.44**	**4.07**
GABARAPL1 down-regulation	Disruption of the intracellular transport of receptors and the autophagy pathway	Low GABARAPL1 expression was correlated with a high risk of metastasis	Metastasis	^87^	**2.04**	NSC
ATG10 up-regulation	Acts as an E2-like enzyme that catalyzes the conjugation of ATG12 to ATG5 and increased autophagy	Lymphovascular invasion	Metastasis	^88^	**2.27**	**5.05**
RAB24 over-expression	Promote the EMT, adhesion and vasculogenic effects	Promotes the malignant phenotype	Tumor growth, metastasis, EMT activation	^88^	**2.76**	**2.23**
ATG5 frameshift mutations	Features of cancers with microsatellite instability (MSI)	Common in gastric and colorectal carcinomas	Tumor development by autophagy deregulation	^64^	**2.63**	**2.94**
CTSB	Dormancy hub gene	Strong biomarker for GBM patient’s survival	Tumor progression	^89^	**10.98**	**9.68**
			Metastasis			
CXCR4 activation	Independent prognostic factor for disease relapse and survival in acute myeloid leukemia (AML) patients	Increases autophagic activity and decreases	Survival	^67,90^	NSC	**3.64**
			Colony formation			
		Cytarabine-induced apoptosis	Proliferation			
IGF-1 activation	Activated protein kinase B (AKT)	Inhibit autophagy	Induce apoptosis in drug resistant cells	^91,92^	**2.80**	**2.05**
INS over-expression	Precursor of insulin	Insulin signaling and the regulation of autophagy are relevant to neurodegenerative disorders	Survival	^93^	**7.81**	**15.51**
MAPK8	Integration point of proliferation, differentiation, transcription regulation and development	Indispensable for TNF superfamily 10 (TNFSF)-induced autophagy	Tumor promotion	^94^	**2.96**	NSC
			Survival			
PIK3CG	Catalytic subunit of class I PI3Ks	Up-regulated under stress conditions	Cell remodeling and tissue failure	^95^	**9.42**	**71.92**
MAPK14	Activation of MAPK14 impairs autophagosome–lysosome fusion	Phosphorylates ATG5 at threonine 75	Survival promoting autophagy	^96,97^	**2.05**	**2.23**
			Cell proliferation			
			Migration, Resistance to apoptosis			
RPS6KB1	In response to mTOR	Autophagy inhibition	Promote protein synthesis	^98^	NSC	**2.08**
			Cell growth			
			Cell proliferation			
DRAM2	Is a lysosomal protein	DRAM2 overexpression induced cell migration proteins including RAC1, RHOA, RHOC, ROCK1, and decreased RHOB expression	Metastasis	^99,100^	**2.05**	NSC
			Proliferation			
			Migration			
			Cell cycle activation			
LAMP1	LAMP1 is lysosomal marker	LAMP1 overexpression reversed the antitumor effects of UBL4A in pancreatic cancer	Cell proliferation	^101,102^	**3.64**	**3.08**
			Migration			
			Invasion			
mTOR	Key regulator of protein synthesis via 4EBP1 and p70S6K1/2 phosphorylation	Increases the translational capacity of cancer cells	Autophagy inhibition	^103^	**6.04**	**6.48**
			Dormancy			
			Metastasis			
RPLP0	Belongs to the L10P family of ribosomal proteins	Affected p21 expression	Cell promotion	^104,105^	NSC	**619.5**
		Induction	Autophagy induction (Survival) in response to RPLP deficiency stress			
		G1 arrest of gastric cancer cells				
CTSS	Is a lysosomal cysteine protease that may participate in the degradation of antigenic proteins	Cleaves some extracellular matrix (ECM) proteins	Tumorigenesis stimulation	^106,107^	**11.35**	NSC
			Metastasis			
ESR1	Point mutations on ESR1 are drivers for resistance, and promote of ERα without the bound ligand	Ligand independently ER stimulation	Proliferation	^108,109^	**9.68**	**4.16**
			Long-distance metastasis			
Autophagy manipulation	eIF4E/eIF4GI knockdown	The decrease in ERα, SMAD5, NFkB, CyclinD1, c-MYC, and HIF1α	The decrease in EMT promoter	^59^	**1049.1**	**1904**
			Increase in EMT inhibitors			
			The decrease in migration capability			

Significant values are in bold. *NSC* non significant change.

## Discussion

According to our findings, the induction of autophagy using AuNRs seems to be completely dependent on the generation of sudden intracellular temperature. The induction of autophagy at two temperatures (43 °C and 48 °C) resulted in completely different consequences. Based on our findings, the BSA–AuNR complex with NIR to heat conversion efficiency and good biocompatibility to 30 ppm could be pretty well candidates for photothermal therapy. Also, an increase of intracellular temperature up to 43 °C caused clean cell death (apoptosis) compared to higher temperatures (48 °C) (necrotic). As we mentioned earlier, the type of cell death has a profound effect on the fate of other cells located in the tumor. Interestingly, LC3 expressing apoptotic cells enhanced by increasing the temperature from 43 to 48 °C which shows autophagic cell death (Fig. 5D, 6B). These data showed that the treatment of human cancer cells with BSA–AuNRs plus PTT could alter the number of cells entering apoptosis. Based on our best knowledge this is the first report about temperature-dependent autophagic cell death by PTT.

For the detailed address of this behavior, we showed that of 86 genes associated with autophagy, the expression of 54 genes was changed in response to temperature variation. Up to now, it has been confirmed that changes in the expression profile of autophagy-related genes contributed to the fate of cancer cells like cell death type, metastasis ability, colony formation, invasiveness, and dormancy which are summarized in (Tables 1, 2, 3). Also, Lu et al. showed that the expression intensity or level of autophagy-related genes affects the cell fate^1^. Interestingly, our results illustrated that the intensity and pattern of these changes were different between 43 and 48 °C (Tables 1 and 2, Supp. Data). In addition, the pattern of some expressed genes is in contrast to each other in two different temperatures like AMBRA1, ATG9B, LC3-I, MAP1LC3B, Atg5, Atg4c, and Atg16. As aforementioned, these findings added a notion that changes in the autophagy effectors contributing to autophagy levels which resulted in altering cell behavior and fate. Furthermore, we showed for the first time that the CMA pathway^2^, which is controlled by some of the heat shock proteins like HSPA8 is activated at 48 °C.

**Table 3:** Autophagic genes involved in cell death (autophagic or apoptotic) VS our findings.

Gene expression changes	Cellular outcome	Effect(s)	Consequences on tumor fate	References	Our study findings	
					43 °C	48 °C
Dormancy activation	P53 overexpression induced by Cdkn1b	Pentose phosphate pathway destruction	Cell death	^110^	**3.24**	NSC
		Increased ROS	Dormancy induction by IFN-b			
FasL (CD95L or CD178), TRAIL and TNF-α activation	DISC formation	Caspase-3, 6 and 7 activation	Directly cell death	^111^	**408.7**	**151.6**
		Bid change into tBid	Mitochondria dependent apoptotic cell death		**4.07**	− **4.10**
					**4.10**	− **2.90**
Autophagy inhibition	ATG7 depletion	Accumulation of damaged mitochondria	The killing of dormant cells	^112^	**2.17**	NSC
		Increase of ROS	Does not affect cell metastasis and proliferation			
		Increase of apoptosis				
Autophagy activation	TMEM166 overexpression	High LC3II/LC3I	Autophagy and apoptosis regulator (autophagic and apoptotic cell death)	^113^	**2.27**	**295.1**
		Vacuolization				
		Mitochondria membrane permeabilization				
GABARAPL2/ULK upregulation	Necessary to maturation of two layers membranous vesicles	Shrinkage of tumor volume in complex with ULK2	Apoptotic cancer cell death	^114^	**3.22**	**2.12**
IRGM KO	p47 dependent GTPase	Negative regulation of IFN signaling	Inhibition of autophagic cell death	^115^	**46.37**	NSC
ATG16L2	Methylation of ATG16L2	Downregulation of autophagy	Autophagic cancer cell death	^116,117^	**29.55**	**21.63**
			Patients survival			
Inherent ATG5 or autophagy KO	Autophagy deficiency	–	Increase apoptotic cell death	^118^	**2.63**	**2.94**
Increase of Bax and Bak1	Intrinsic pathway of apoptosis (mitochondria)	Indirectly effect on autophagy by inactivation of BaK1 and Bax	Increase cancer cell apoptosis	^119,120^	**492.85**	**299.21**
Bid and PUMA (apoptosis-associated genes)	BID acts as molecular link between apoptosis and autophagy	Contribute to identifying the molecular mechanism by which autophagy drives cells to death	Autophagic cell death	^66,121^	**3.35**	**2.42**
	PUMA is certain substrate for CMA					
DAPK1	One of the most important genes in intra/extra cellular apoptotic pathways	ARHI dependent	Tumor suppressor	^65^	**2280.29**	**1453.18**
			Apoptotic cell death			
PTEN	Negative regulator of PI3K/AKT/mTORC1	Autophagy activation, PI3K/Akt inhibition, PI3K/AKT/mTORC1 inhibition	Tumor suppressor	^47^	**35.38**	**23.02**
PTEN	Indirectly positive autophagy regulator	PTEN inhibitors (Tsc1 or Tsc2, p27) and Foxo3a	Escape from dormancy	^122^	**35.38**	**23.02**
PTEN	Tumor suppressor	Apoptosis modulators DRAM, DAPk and DRP-1, PTEN, E93, Akt/PKB, and mTOR), Bcl-2 family proteins, TRAIL and beclin 1	Autophagy act as upstream control of apoptosis death	^113^	**35.38**	**23.02**
GAA deficiency	Lysosomal hydrolysis of glycogen to glucose (glycogen storage disease II, or Pompe)	Accumulation of abnormal proteins and organelles due to inhibition of autophagy	Cell death	^123^	NSC	**3.69**
Autophagy abortion	DRAM1 overexpression	By p53	Apoptotic death	^124^	**4.36**	**2.65**

Significant values are in bold. *NSC* non significant change.

All together molecular, transcriptional, and colony formation experiments in this work demonstrated that the low level of mild hyperthermia (43 °C) has protective effects on the cancer cells, while the higher level of mild hyperthermia (48 °C) led to cytotoxicity in SH-SY5Y cells. Therefore, despite the induction of more apoptosis, our findings suggest that autophagy stimulation by PTT at 43 °C leads to increased autophagic survival and dormancy. The findings are consistent with the results obtained from the study of genes involved in autophagy in this study, which finally can threaten the patients’ lives in the clinic.

The most important finding in this study was the rethinking of the usage of autophagy inhibitors in the PTT therapeutic regimens. Colony formation assay findings as the outcome of PTT were shown that, despite the loss of a large number of cells at 48 °C, cells can form colonies in the presence of autophagy inhibitor (Fig. 7A–E). It means that using autophagy inhibitors in lower temperatures can be beneficial, while in higher temperatures cause to exacerbate the fate of resistant tumor cells. It seems that a higher level of autophagy (e.g., EIF4E and eIF4G) and released factors from neurotic cells mutually evoked the invasion and metastasis-related signaling pathways at 48 °C (Tables 1, 3). For instance, it has been shown that the tumor formation process that arises from the Ras pathway activation requires high levels of autophagy for survival and cell proliferation^3^.

Since the use of hyperthermia to eliminate cancers has entered into the clinical phase, the results of this study allied to the others^4^ ring the alarms for all of us, which forcefully recommended using all necessary experiments to prevent unwanted complications. As shown in this study, after eradication of a large number of cancer cells at 48 °C, the remaining cells in the presence of an autophagy inhibitor will be able to form a tumor. This finding is in line with other scientists who have previously shown that even 100 cancer stem cells are enough to form a tumor^5^. So, we strongly suggest further investigations to find the relationship between autophagic gene machinery and PTT-induced autophagy.

## Methods

### Materials

HAuCl_4_, Hydroxychloroquine (HCQ), and 3-MA were obtained from Sigma-Aldrich and dissolved in deionized water to reach 100 mM, 1 mM, and 100 mM stock solutions, respectively. The antibodies used included LC3-II (Miltenyi Biotech, 130-090-853). Albumin, cetyltrimethylammonium bromide (CTAB), AgNo3, ascorbic acid, and NaBH4 were purchased from Sigma-Aldrich. The human neuroblastoma cell line SH-SY5Y was obtained from the Stem Cell Research Center, Tabriz University of Medical Sciences. The high-glucose Dulbecco’s Modified Eagle’s Medium (DMEM/HG), fetal bovine serum (FBS), l-glutamine, 0.25% Trypsin–EDTA, and Penicillin/Streptomycin (Pen-Strep) were all purchased from Gibco. The Annexin-V-FLUOS Staining kit was purchased from Roche Company.

### Characterization

UV–Vis spectra were recorded on a spectrophotometer (Cecil UV, UK). Transmission electron microscopy (TEM) analyses were performed by a Zeiss EM900 80 kV electron microscope. TEM samples were prepared by dropping a small quantity of dispersion onto formvar carbon 300 mesh thick grids. The size of AuNRs was determined by the dynamic light scattering (DLS) technique using Zetasizer Nano ZS90; Malvern Instruments, UK. The samples were irradiated with a laser model MDL-III—at a wavelength of 808 nm and the energy of 2.5 W (Changchun New Industries Optoelectronics Tech. Company). The dispersions’ temperature was monitored using a digital thermometer with a thermocouple probe from Pyrometer Instrument Company. In the MTT assay, the absorbance (OD) of samples was measured at 570 nm using a microplate reader (BioTek, USA). Flow cytometry tests were performed on a flow cytometer (Partech space flow^®^), and data were analyzed by FlowJo software (version V.10).

### Gold nanorods (AuNRs) synthesis

The synthesis of AuNRs was done according to the previously described seedless growth method^26^. Briefly, 5 mL of 1.0 mM HAuCl_4_ was added to 5 mL of 0.20 M CTAB. Then, 250 mL of 4.0 mM AgNO_3_ was added, and the solution was gently shaken. By adding 8 mL of 37% HCl pH of the solution was adjusted to 1–1.15. Subsequently, 70 mL ascorbic acid (78.8 mM) was added to the solution then gently shaking until the solution was clear. After that, 15 mL of 0.01 M ice-cold NaBH_4_ was injected to the unstirred growth solution and allowed to react for 6 h. The solution was centrifuged at 10,000 rpm for 15 min, and the supernatant was removed. The pellet was suspended in water and centrifuged at the same speed for an additional 15 min.

### Surface modification of AuNRs with BSA (BSA–AuNRs)

BSA was used to modify the surface of the AuNRs according to a method described by Tebbe et al.^48^. Briefly, the NP dispersions were diluted with deionized water and slowly added to the BSA solution under ultrasonication (BSA solution/NP dispersions, 1:1 v/v). The BSA solution contains BSA (10 mg/mL), and 0.02% citrate and pH were adjusted to 7. The NPs were sonicated for 30 min and then centrifuged at 6500 rpm for 6 min followed by replacement of supernatant with 10X-diluted BSA solution (1 mg/ml, pH = 12, 0.02% citrate). The final solution was stirred for at least 24 h. Eventually, the particles were centrifuged and washed with deionized water before use.

### Temperature elevation modeling

After determining the cellular uptake rate of nanoparticles, to simulate the uptaken nanoparticles’ ability to increase the ambient temperature, we dissolved the same amount of BSA–AuNRs in deionized water and irradiated with 808-nm continuous-wave NIR laser at different power densities. The temperature of samples was monitored using a digital thermometer with a thermocouple probe.

### Cell culture

The human neuroblastoma cell line, SH-SY5Y cells, were cultured in DMEM/HG supplemented with 10% FBS and 1% Pen-Strep. The culture flasks were maintained at 37 °C under 5% CO_2_ under a humidified atmosphere. Cells at passages 3 to 6 were used in this study. The cells were sub-cultured using 0.25% Trypsin–EDTA solution.

### Analyzing cellular uptake of AuNRs using atomic absorption spectroscopy (AAS)

SH-SY5Y cells were seeded in 6-well plates with at an initial cell density of 2 × 10^5^ cells/well. After reaching 70–80% confluence, the culture medium was replaced with fresh medium containing 30 ppm BSA–AuNRs and 2% FBS. The culture medium was discarded after a 4-h incubation period. Then, the cells collected using an enzymatic solution (Trypsin–EDTA) dissolved in Aqua Regia overnight and heated to about 140 °C to excluding hydrogen chloride and nitrogen oxides until the solution became colorless and clear^49^. After that, the digested solution incubated in an aqueous solution containing 2% nitric acid and 1% hydrogen chloride for loosing and detaching gold atoms in nanoparticle lattice; the total cellular gold content was determined by AAS.

### AuNR-mediated PTT protocol in vitro

In this study, SH-SY5Y cells were randomly allocated into three different groups as follows: Control, 30 ppm BSA–AuNRs (43 °C), and 30 ppm BSA–AuNRs (48 °C). In this regard, cells were maintained for 4 h, followed by replacement with BSA–AuNR free culture medium. Then, cells were irradiated by an 808-nm NIR laser at a power density of 0.3 and 0.9 W for 8 min (to reaching the 43 °C and 48 °C) in the absence and presence of 10 µM HCQ. During laser irradiation, the temperature of the culture medium was carefully monitored.

### MTT assay

The localized cell-killing ability of PTT at the varied densities (0.3 and 0.9 W) was assessed by a typical MTT assay. After irradiation, cells were maintained for the next 24 h, and the survival rate was measured by using a standard MTT assay. To this end, the culture medium was discarded, replaced with 5 mg/ml MTT solution, and incubated at 37 °C for 3–4 h. Thereafter, the supernatants were removed, and 100 µl DMSO solution was added to each well to dissolve formazan crystals. Percent of cell viability was denoted as the relative absorbance of treated versus untreated viable cells. The following formula was used to calculate the inhibition of cell growth:

$$ {\text{Cell}}\;{\text{ viability}}\;(\% ) = \left( {{\text{mean}}\;{\text{ of }}\;{\text{the}}\;{\text{ absolute }}\;{\text{value}}\;{\text{ of}}\;{\text{ treatment}}\;{\text{ group/mean }}\;{\text{of}}\;{\text{ the}}\;{\text{ absolute }}\;{\text{value }}\;{\text{of}}\;{\text{ control}}} \right) \times 100\% . $$

### Flow cytometric analysis of apoptotic cells

To quantify the effect of PTT at varied energy densities on SH-SY5Y cells, we performed flow cytometry analysis. To this end, cells were cultured in each well of 24-well plates and irradiated as above-mentioned. Twenty-four hours post-PTT, cells were harvested, permeabilized, and stained using the Annexin-V-BioXBio Staining kit according to the manufacturer’s instructions. The percent of alive, apoptotic, and necrotic cells were analyzed by the Partech Cyflow space^®^ Flow cytometry system. The raw data were processed using FlowJo software (version V.10).

### Monitoring autophagy status using flow cytometry and immunofluorescence staining

#### Flow cytometry analysis

To assess autophagy status after PTT, the SH-SY5Y cells were incubated with the FITC-conjugated LC3 antibody. Twenty-four hours after PTT, cells were collected from different groups, washed twice with PBS, and fixed using pre-cooled 3.7% paraformaldehyde for 8 min. By using the permeabilization buffer, cells were permeabilized and blocked with 1% BSA. Then, cells were incubated in a solution containing a diluted FITC-conjugated LC3 (1:200) antibody overnight. After twice PBS washes, cells were incubated with DyLight 488 conjugated secondary antibody (1:500) for 1 h. Finally, the samples were analyzed by Partech Cyflow space^®^ flow cytometry and FlowJo software (version V.10).

### Immunofluorescence imaging

Cells (1 × 10^4^) were seeded in each well of 8-well Chambered Cell Culture Slide (SPL) and cultured until reaching 70–80% confluency. Here, the cells were classified into Control and AuNRs groups. In the AuNRs group, cells were exposed to 808-nm laser with different energy densities of 0.3 and 0.9 W for 8 min to reach temperatures of 43 °C and 48 °C. After 12 h, cells were washed with PBS and fixed with 3.7% paraformaldehyde for 10 min. Fixed cells were permeabilized with permeabilization buffer for 15 min, blocked with 1% BSA for 60 min, and incubated with anti-LC3 antibodies (1:200) overnight. After washing with PBS, cells were incubated for 1 h with DyLight 488 conjugated secondary antibody (1:500). Finally, cells were evaluated under a fluorescence microscope (Olympus microscope Bh2-RFCA, Japan). The images were analyzed using the Leica Application Suite 2.02 software.

### PCR Profiler array analysis of various gene expression related to autophagy

The Human Custom RT2 Profiler™ PCR Array (CAPH11870A, Qiagen) profiles the expression of 84 genes involved autophagy. RNA was purified using the Qiagen RNAse kit, including on-column DNAse treatment to remove genomic DNA. cDNA was prepared with the RT2 First Strand Kit (SA Biosciences, Frederick, Maryland, USA). A PCR profiler array was performed (RT2 SYBR Green/ROX qPCR Master Mix; SA Biosciences) in 96-well plates on an ABI 7300 instrument (Applied Biosystems, California, USA). For data analysis, the ΔΔCt method was applied using the RT2 Profiler PCR Array software package, and statistical analyses performed (n = 3). This package uses ΔΔCT–based fold change calculations and the Student’s *t* test to calculate two-tail, equal variance P-values. The fold changes were calculated using the equation 2^−ΔΔCt^. If fold change was greater than 1, the result was considered as fold up-regulation. If fold change was less than 1, the negative inverse of the result was considered as fold down-regulation^34^.

### Spheroid-formation assay

The possible effect of BSA–AuNR-mediated PTT was investigated on the clonogenicity property of SH-SY5Y cells. In brief, cells were exposed to 808-nm NIR laser and collected using the enzymatic solution. Thereafter, 1000 cells from the abovementioned groups were resuspended in a solution containing 0.1% agar, 1% gelatin, and 2.5% methylcellulose and transferred into each well of 6-well plates in the presence and absence of 10 μM HCQ. The plates were kept for 21 days, and the number of colonies was calculated per each well. This assay was done in triplicate.

### Monitoring the levels of LC3, P62, and Ki67 using ELISA

After completion of laser irradiation, cells were collected and lyzed using protein lysis buffer (50 mM NaCl, 0.1% SDS, 50 mM Tris–HCl, 2 mM EDTA, 1% NP-40). The samples were centrifuged at 14,000 rpm at 4 °C, supernatants collected and stored at − 20 °C until use. To perform ELISA assay, 1 µg/ml of P62 (Abcam), LC3 (Abcam), and Ki67 (Abcam) antibodies were poured in each well of 96-well polystyrene plates and incubated at 4 °C overnight. The next day, supernatants were discarded, and wells were blocked using 1% BSA solution at room temperature for 30 min. We added 1 µg/ml of protein from different groups and plates were incubated for 1 h at room temperature. After twice PBS wash, HRP-conjugated secondary antibody was added to each well and maintained at room temperature for 1 h. Following PBS wash, 3, 3′-Diaminobenzidine (DAB) was added, and the reaction stopped using 5% H_2_SO_4_. Finally, the OD was read at 450 nm using a microplate reader.

### Statistical analysis

Data were presented as mean ± SD. For the comparison of multiple groups, One-Way ANOVA and Tukey post hoc was used. P values < 0.05 were considered statistically significant. All experiments were done in triplicate otherwise mentioned.

## Supplementary Information

Supplementary Figures.
