# Supplementary material for: Mild hyperthermia induced by gold nanorods acts as a dual-edge blade in the fate of SH-SY5Y cells via autophagy
Source: Sci Rep. 2021 Dec 14;11:23984. doi: 10.1038/s41598-021-02697-y (PMC8671444; doi:10.1038/s41598-021-02697-y)
Supplement: Supplementary file 1 — Supplementary Figures. [file 41598_2021_2697_MOESM1_ESM.docx]

**Mild hyperthermia induced by gold nanorods acts as a dual-edge blade in the fate of SH-SY5Y cells via autophagy**

Maryam Ghafarkhani ^a^, Cigir Biray Avci ^b^, Reza Rahbarghazi ^c,d^, Abbas Karimi ^e^, Majid Sadeghizadeh ^f^, Amir Zarebkohan ^a,d†^, Farhad Bani ^a,d‡^,

^a^ Department of Medical Nanotechnology, Faculty of Advanced Medical Sciences, Tabriz University of Medical Sciences, Tabriz, Iran

^b^ Department of Medical Biology, Medical Faculty, Ege University, Bornova, 35100, Izmir, Turkey

^c^ Department of Cellular and Molecular Biology, Faculty of Advanced Medical Sciences, Tabriz University of Medical Sciences, Tabriz, Iran

^d^ Stem cell research Center, Tabriz University of Medical Sciences, Tabriz, Iran

^e^ Department of Molecular Medicine, Faculty of Advanced Medical Sciences, Tabriz University of Medical Sciences, Tabriz, Iran

^f^ Department of Nanobiotechnology, Faculty of Medical Sciences, Tarbiat Modares University, Tehran, Iran

***Corresponding Authors:***

1. **Dr. Amir Zarebkohan**

Department of Medical Nanotechnology, Faculty of Advanced Medical Sciences, Tabriz University of Medical Sciences, Tabriz, Iran.

Email: [zarebkohana@tbzmed.ac.ir](mailto:zarebkohana@tbzmed.ac.ir)

Post Code: 516661-4733
Tell: (+98-41) 3355790

Mobile: (+98-990) 544 9299

1. **Dr. Farhad Bani**

Department of Medical Nanotechnology, Faculty of Advanced Medical Sciences, Tabriz University of Medical Sciences, Tabriz, Iran.

Email: [banif@tbzmed.ac.ir](mailto:banif@tbzmed.ac.ir)

Post Code: 516661-4733
Tell: (+98-41) 3355790

**Supplementary Figure 1.** Analysis of all 86 genes expression profile. Genes linking autophagosome to lysosome.

**(48 ^o^C fold change 43 ^o^C fold change )**

**Supplementary Figure 2.** Analysis of all 86 genes expression profile. Genes involved in autophagic vacuole formation.

**Supplementary Figure 3.** Analysis of all 86 genes expression profile. Genes responsible for protein targeting to membrane/vacuole.

**Supplementary Figure 4.** Analysis of all 86 genes expression profile. Genes responsible for protein transport.

**Supplementary Figure 5.** Analysis of all 86 genes expression profile. Genes involved in protein ubiquitin.

**Supplementary Figure 6.** Analysis of all 86 genes expression profile. Genes with protease activity.

**Supplementary Figure 7.** Analysis of all 86 genes expression profile. Co-regulators of autophagy and apoptosis.

**Supplementary Figure 8.** Analysis of all 86 genes expression profile. Co-regulators of autophagy and apoptosis.

**Supplementary Figure 9.** Analysis of all 86 genes expression profile. Autophagy induction by intracellular pathogens.

**Supplementary Figure 10.** Analysis of all 86 genes expression profile. Autophagy in response to other intracellular signals.

**Supplementary Figure 11.** Analysis of all 86 genes expression profile. Chaperone-mediated autophagy.

**Supplementary Figure 12.** Analysis of all 86 genes expression profile. Other important genes related to autophagy.
